# Genetic and treatment profiles of patients with concurrent Epidermal Growth Factor Receptor (EGFR) and Anaplastic Lymphoma Kinase (ALK) mutations

**DOI:** 10.1186/s12885-021-08824-2

**Published:** 2021-10-15

**Authors:** Xiaodan Yang, Jia Zhong, Zhuo Yu, Minglei Zhuo, Min Zhang, Rongrong Chen, Xuefeng Xia, Jun Zhao

**Affiliations:** 1 Key laboratory of Carcinogenesis and Translational Research (Ministry of Education), Department of Thoracic Medical Oncology, Peking University Cancer Hospital & Institute, No.52, Fucheng Road, Haidian district, Beijing, China; 2 Department of Medical Oncology, National Cancer Center/National Clinical Research Center for Cancer/Cancer hospital, Chinese Academy of Medical Sciences and Peking Union Medical College, Beijing, China; 3 Beijing Tsinghua Changgung Hospital, Beijing, 102218 China; 4 GenePlus-Beijing, Beijing, 102206 China

**Keywords:** EGFR mutation, ALK rearrangement, Non-small cell lung cancer

## Abstract

**Background:**

EGFR and ALK alternations often contribute to human malignancies, including lung cancer. EGFR and ALK mutations are usually sensitive to EGFR-tyrosine kinase inhibitors (TKIs) and ALK-TKIs. Although generally mutually exclusive, these mutations do co-exist in rare cases. This study investigated the frequencies, clinical characteristics, therapeutic efficacies, and genetic profiles of lung cancer patients with EGFR and ALK co-mutations.

**Methods:**

Patients with concurrent EGFR and ALK mutations were included in this study, which analyzed mutation profiles and treatment histories. SPSS20.0 were used for survival analysis.

**Results:**

Among 271 ALK-positive (ALK-pos) and 2975 EGFR-positive (EGFR-pos) patients in our database, nine (2.6% of ALK-pos and 0.2% of EGFR-pos) patients had concurrent EGFR and ALK mutations (including three exon19 Indel + EML4-ALK, two exon19 Indel + STRN-ALK, two L858R + L1152R, one L858R + EML4-ALK, and one G719C + S768I + STRN-ALK). Eight patients had at least one type of EGFR-TKIs treatment. The median progression free survival (PFS) of these patients on first-generation EGFR-TKIs was 14.5 months (95% CI: 11 - NR). Of these eight patients, one who progressed on Gefitinib and subsequently on Osimertinib had a T790M + C797G. The other seven EGFR-TKIs resistance patients had no known resistance mutations. No patients had ALK mutations before treatment, so ALK mutations may have developed as resistance mechanisms during EGFR-TKIs therapies. EGFR-TKIs-treated patients with EGFR/ALK L1152R mutations generally had a shorter PFS than patients with other mutation combinations.

**Conclusions:**

ALK and EGFR mutations coincide at a relatively low frequency in lung cancer patients. ALK mutations developed either synchronously or heterochronously with EGFR mutations. Two ALK mutations (L1152R and STRN-ALK) may co-exist with EGFR mutations at a higher frequency than others. Most EGFR/ALK co-alteration patients (other than the EGFR/ALK L1152R type) can benefit from first line EGFR-TKIs.

**Supplementary Information:**

The online version contains supplementary material available at 10.1186/s12885-021-08824-2.

## Background

Lung cancer has a high morbidity and mortality worldwide [1]. Historically, chemotherapy and radiotherapy are the main treatments for patients with metastatic lung cancer, and the five-year survival rate remains low (< 15%) [2]. However, with the discovery of driving genes, targeted therapy has significantly improved the prognosis of non-small cell lung cancer (NSCLC) [3–5].

EGFR mutations and ALK rearrangements are the two most important driving genes for NSCLC. Of these, EGFR mutations are the most common operable driver mutations found in NSCLC patients, occurring in approximately 10% of white patients, and up to 50% of Asian patients [6–8]. In a series of studies, patients with EGFR mutations accepted EGFR-TKIs, and the progression-free survival (PFS) was superior to chemotherapy [9]. The occurrence of an ALK rearrangement is 3 to 5% of patients with NSCLC. They often have unique clinical and pathological characteristics including a younger age, no history of heavy smoking, and adenocarcinoma [10–13]. ALK kinase inhibitors are effective therapies with in vitro and in vivo models, as well as with NSCLC patients with ALK rearrangements [5, 10, 14]. Mutations in driving oncogenes are mostly independent (mutually exclusive), overlapping in only 3 to 5% of the cases [15]. However, with the application of Next-generation sequencing, more low-frequency mutations, as well as concurrent mutations can be detected.

Because of the rarity of concurrent mutation, there is limited knowledge of their prognostic and predictive value. Takaaki et al. suggested that new alterations in ALK or EGFR may result from drug resistance mechanisms against EGFR or ALK tyrosine kinase inhibitors (TKIs) [16].

In our study, we measured the frequency of EGFR and ALK concurrent mutations associated with lung cancer, and studied the responses to TKI treatment in this rare patient population.

## Methods

### Patients

We screened for concurrent EGFR and ALK mutation status in a multi-center retrospective analysis of 5755 patients with lung cancer from January, 2017 to September, 2018. Among them were 2975 EGFR-positive, and 271 ALK-positive patients. Those with actionable mutations in both ALK and EGFR (detected either simultaneously or subsequently) were included our research cohort. The clinicopathological characteristics, treatment histories, treatment outcomes, and survival information of these patients were collected. Mutations of ALK and EGFR filtered with the annotated oncoKB levels of evidence. This study was approved by the Ethics Committee of the Peking University Cancer Hospital in Beijing, China. All other centers concurred, and all patients providing tissue or other medical data gave written informed consent in accordance with the Helsinki Declaration.

### Next generation sequencing (NGS)

All tissues samples had at least 20% tumor cell content and histologic classification were confirmed by pathologist. Nucleic acid sequencing was performed on Illumina Nextseq CN 500 or Gene+Seq 2000 in Geneplus-Beijing which certified by College of American Pathologists [17, 18]. Genomic tumor DNA was extracted from formalin-fixed paraffin-embedded tumor tissues, plasma and white blood cell using QIAamp DNA mini kit (Qiagen, Valencia, CA), QIAamp Circulating Nucleic Acid Kit (Qiagen) and DNeasy Blood Kit (Qiagen), respectively. And sequencing libraries were prepared using the KAPA DNA Library Preparation Kit (Kapa Biosystems, Wilmington, MA, USA), and Illumina TruSeq DNA Library Preparation Kits (Illumina, San Diego, CA). Targeting sequencing region including ~ 1.4 Mbp of 1021 cancer-related genes as well as ~ 230 Kbp of 59 genes (Supplementary Table 1). Gene+Seq2000 was approved by the National Medical Products Administration (NMPA) in August 26, 2019. It is used for clinical DNA sequencing of human samples to detect sequences which may lead to disease or susceptibility. Gene+Seq2000 utilizes a state-of-the-art core technology called DNBSEQ. It has the same mechanism as MGI sequencing platforms, which has a comparable performance to Illumina NovaSeq 6000 [19]. Gene+Seq2000 has high accuracy with 99.69% on human genomic sequencing. The sensitivity and specificity of Gene+Seq2000 were 100% in 269 lung cancer patients who had known driver mutations on EGFR/KRAS/ALK. It has also demonstrated good performance on clinical practice and research [20–22].

Nextseq CN500 was approved by National Medical Products Administration (NMPA) in November 7, 2019. It is a high throughput sequencing instrument developed with Illumina based on sequence by synthesis (SBS) technology. It is used for human DNA sequencing to detect gene sequences which may lead to disease or susceptibility. The sensitivity and specificity of Nextseq CN500 were 100% when compared with HiSeq3000 and NovaSeq6000. It also shows good performance on clinical practice and research [23, 24].

Minimal mean effective depth of coverage was 300× in tissue and 1000× in plasma samples. Clean reads which removed which removed adaptor sequences and low-quality reads were aligned to the reference human genome (hg19) by Burrows-Wheeler Aligner (BWA, version 0.7.12-r1039). Realignment and recalibration were performed using GATK (version 3.4–46-gbc02625). Single nucleotide variants (SNV), small insertions and deletions (Indels) and copy number alterations were identified by MuTect (version 1.1.4), NChot, GATK and CONTRA (v 2.0.8) respectively.

### Data interpretation

SNVs, InDels, copy number alterations, and select gene fusions and rearrangements were detected which filtered out synonymous variants, known germline variants in dbSNP, and variants that occur at a population frequency of > 1% in the Exome Sequencing Project. Germline variants were interpreted to five categories following ACMG.

### Evaluations of TKI treatment

The tumors were evaluated during the treatment with EGFR-TKIs to measure the tumor response according to the Response Evaluation Criteria in Solid Tumors (RECIST 1.1). The objective response rate (ORR) categories included complete response (CR), partial response (PR), stable disease (SD) and progressive disease (PD.)

### Statistical analysis

Statistical analyses were performed using SPSS 20.0 software (SPSS Inc., Chicago, IL, USA). The relationships between the EGFR and ALK co-mutation with smoking status was examined using chi-square tests. The Kaplan-Meier was used to estimate the PFS and calculate a 95% confidence interval (CI).

## Result

### Patients with ALK/EGFR actionable mutations

We analyzed 2975 EGFR-positive patients and 271 ALK-positive patients. Different mutations were detected in each patient (Supplementary Table 2). From those, nine EGFR/ALK concurrent mutation patients were identified, accounting for 3.3% (9/271) of ALK-positive patients and 0.30% (9/2975) of EGFR-positive patients. Six patients were synchronous EGFR/ALK co-alteration, and three were heterochronous. All patients were TNM diagnosed with stage IV adenocarcinoma. The ALK and EGFR co-mutation forms included the following: three exon19Indel + EML4-ALK; two exon 19Indel + STRN-ALK; two L858R + L1152R; one L858R + EML4-ALK; and one G719C + S768I + STRN-ALK (Table 1). Of the nine patients with EGFR/ALK co-mutations, five were EGFR19del. Clinical characteristics including smoking status and specific EGFR mutation types were not associated with the co-alteration.

**Table 1:** Characteristics of patients with EGFR/ALK co-alterations

Patients	Age range at diagnosis	TNM stage at diagnosis	Pathological	Metastasis	EGFR type	ALK type
1	50–70	IV	Adenocarcinoma	Node, bone	19del	STRN-ALK
2	50–70	IV	Adenocarcinoma	Multiple metastases	19del	STRN-ALK
3	50–70	IV	Adenocarcinoma	bone	L858R	L1152R
4	50–70	IV	Adenocarcinoma	Unknown	L858R	L1152R
5	50–70	IV	Adenocarcinoma	Brain, bone, liver, node	L858R	EML4-ALK
6	50–70	IV	Adenocarcinoma	Pleura, peritoneum, mesentery	19del	EML4-ALK
7	50–70	IV	Adenocarcinoma	Liver, lung, bone, node	19del	EML4-ALK
8	50–70	IV	Adenocarcinoma	Brain, mediastinum, hilum	G719C + S768I	STRN-ALK
9	50–70	IV	Adenocarcinoma	Multiple metastases	19del	EML4-ALK

### Outcome of EGFR-TKIs treatment in lung Cancer patients with EGFR/ALK co-mutations

**Fig. 1:**
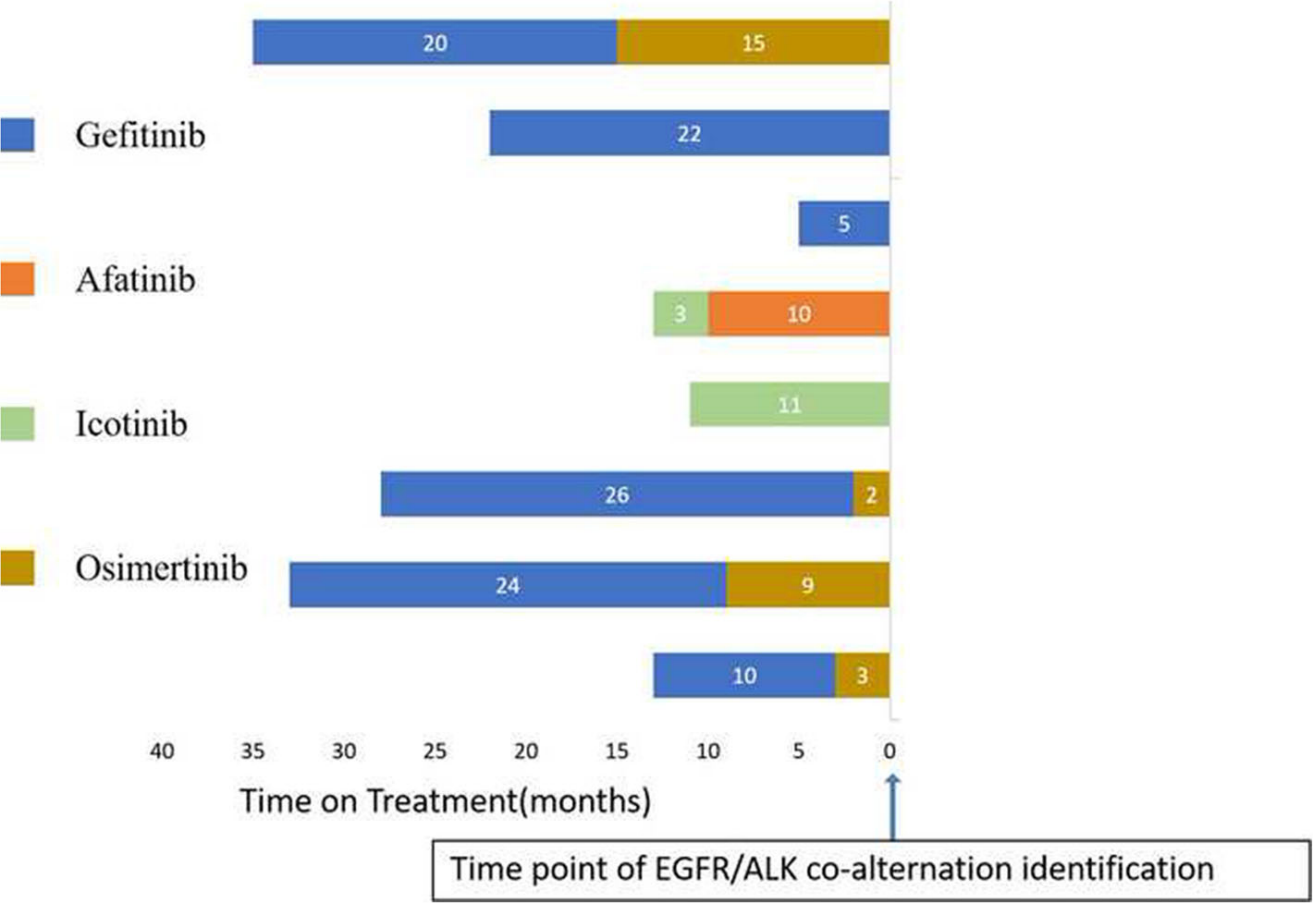
A detailed history of treatment and subsequent clinical outcomes of eight EGFR/ALK co-alternation patients are presented. The number in parentheses represent the PFS (Progression-free survival after EGFR-TKI treatment) for each drug, The arrow presents time point of EGFR/ALK co-alternation identification

**Fig. 2:**
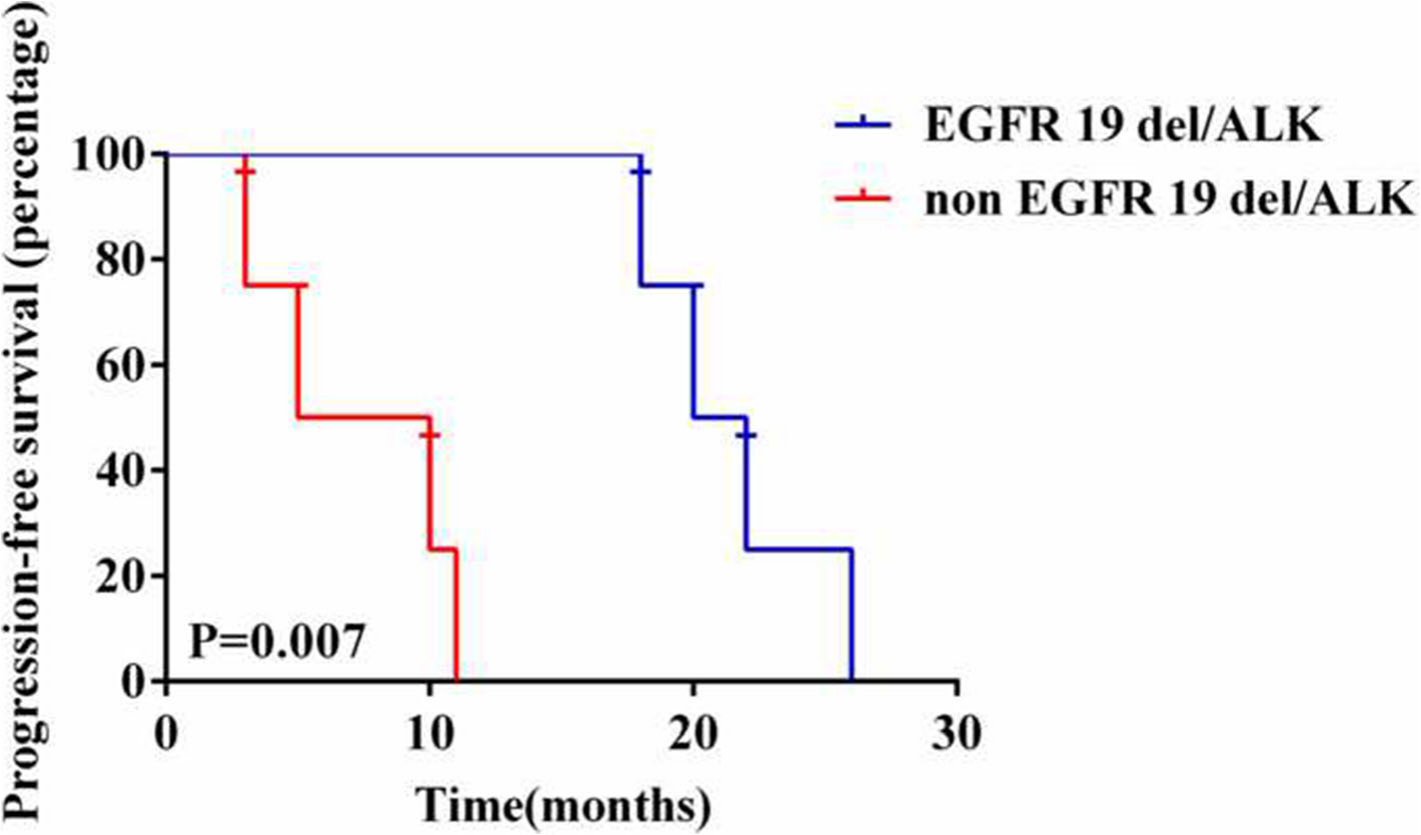
Comparison of PFS between EGFR 19 del/ALK and non-EGFR 19del/ALK on EGFR-TKI (21.5 vs 7.25 months, *P* = 0.007)

**Fig. 3:**
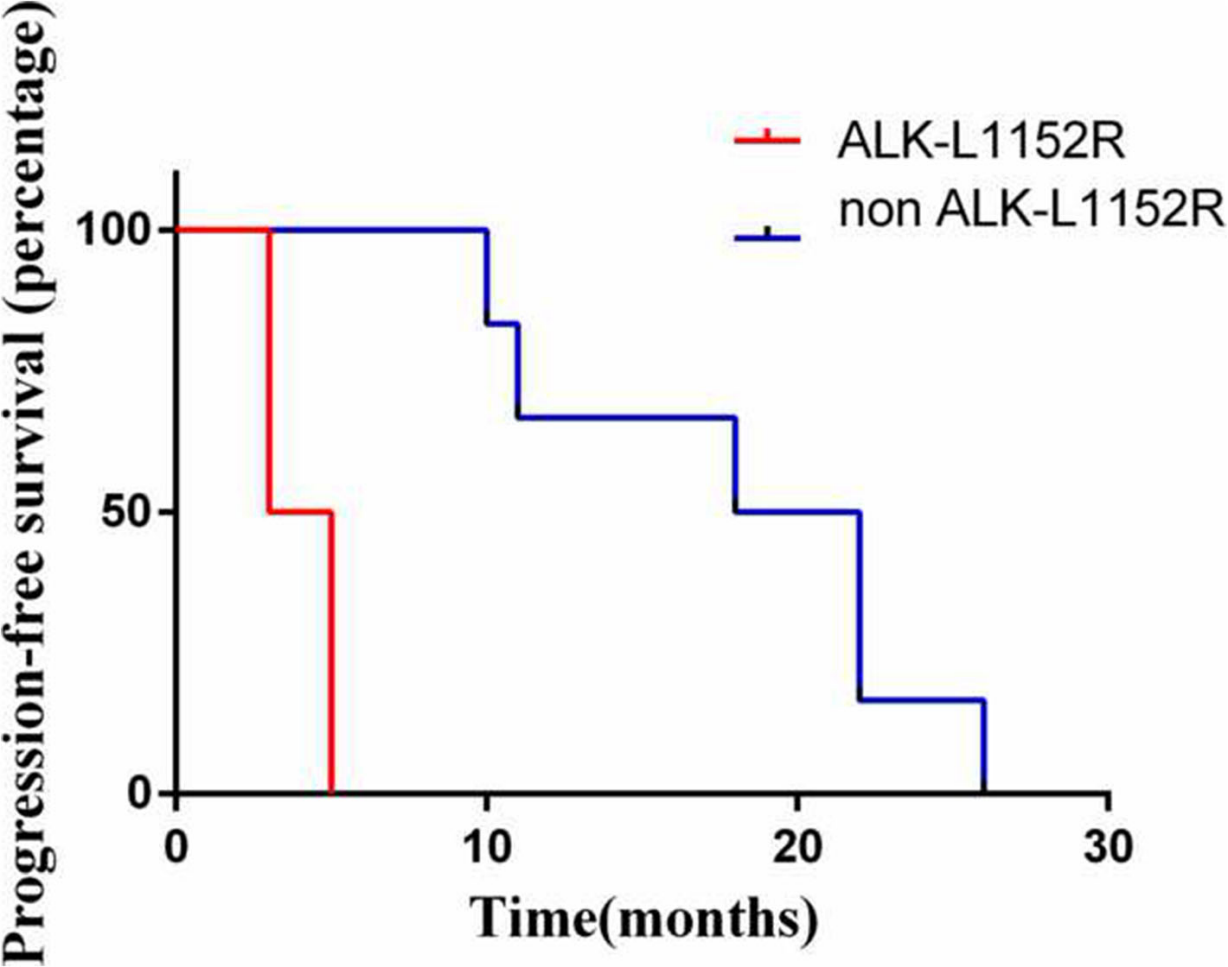
Comparison of PFS between ALK-L1152R/ EGFR and non-ALK-L1152R/ EGFR on EGFR-TKI (18.2 vs 4 months, *P* = 0.004)

We collected clinical information for the nine patients with EGFR/ ALK co-mutations. Eight of nine EGFR/ ALK co-mutation patients received EGFR-TKIs, including Osimertinib (*n* = 4), Gefitinib (*n* = 6), Afatinib (*n* = 1), and Icotinib (*n* = 2). EGFR-TKI was treated as first-line therapy in four patients, second-line therapy in five patients, and third-line therapy in four patients. Five patients received two lines of EGFR TKI, three patients received only one line. The median PFS of these patients on first-line EGFR-TKIs was 14.5 months (95% CI: 11 – NR, Fig. 1). Among the eight EGFR/ALK co-mutation patients receiving EGFR-TKIs, four had the EGFR19del/ALK co-mutation, and four had other co-mutation genotypes. Kaplan-Meier analysis showed that after the first line of EGFR-TKI treatment, the median PFS (mPFS) of patients with EGFR 19del/ALK co-mutation (*n* = 4) was significantly higher than that of patients with non-EGFR19del/ ALK co-mutation (*n* = 4, one patient had unclear PFS information, mPFS of 21.5 vs 7.25 months, *p* = 0.007, Fig. 2). In addition, the mPFS of patients with ALK-L1152R/EGFR (*n* = 2) was significantly shorter than that of patients with other co-mutation genotypes (*n* = 7, one patient had unclear PFS information, mPFS of 18.2 vs 4 months, *p* = 0.004, Fig. 3). Moreover, five patients were treated with EGFR-TKIs after the detection of a co-mutation. In this group the PFS were: two patients at 10 months; and one each at 11, 18, and 26 months, indicating that the treatment with TKI after the co-alternations of EGFR and ALK did not affect the efficacy of TKI.

Cox model analyses for the eight patients (including smoking status, EGFR and ALK mutation types, line of treatment, types of EGFR-TKIs, and concomitant genetic alterations) were included in the initial Cox model. By backward elimination, we found that EGFR mutation types (non-EGFR19del) and ALK rearrangement types (ALK-L1152R) are independent factors that associate with poor PFS in EGFR-TKIs treatment (HR 3.70, 95% CI: 1.76–23.58, *p* = 0.024; and HR 4.67, 95% CI: 1.86–33.09, *p* = 0.015; respectively).

### Potential mechanism of TKI resistance

**Fig. 4:**
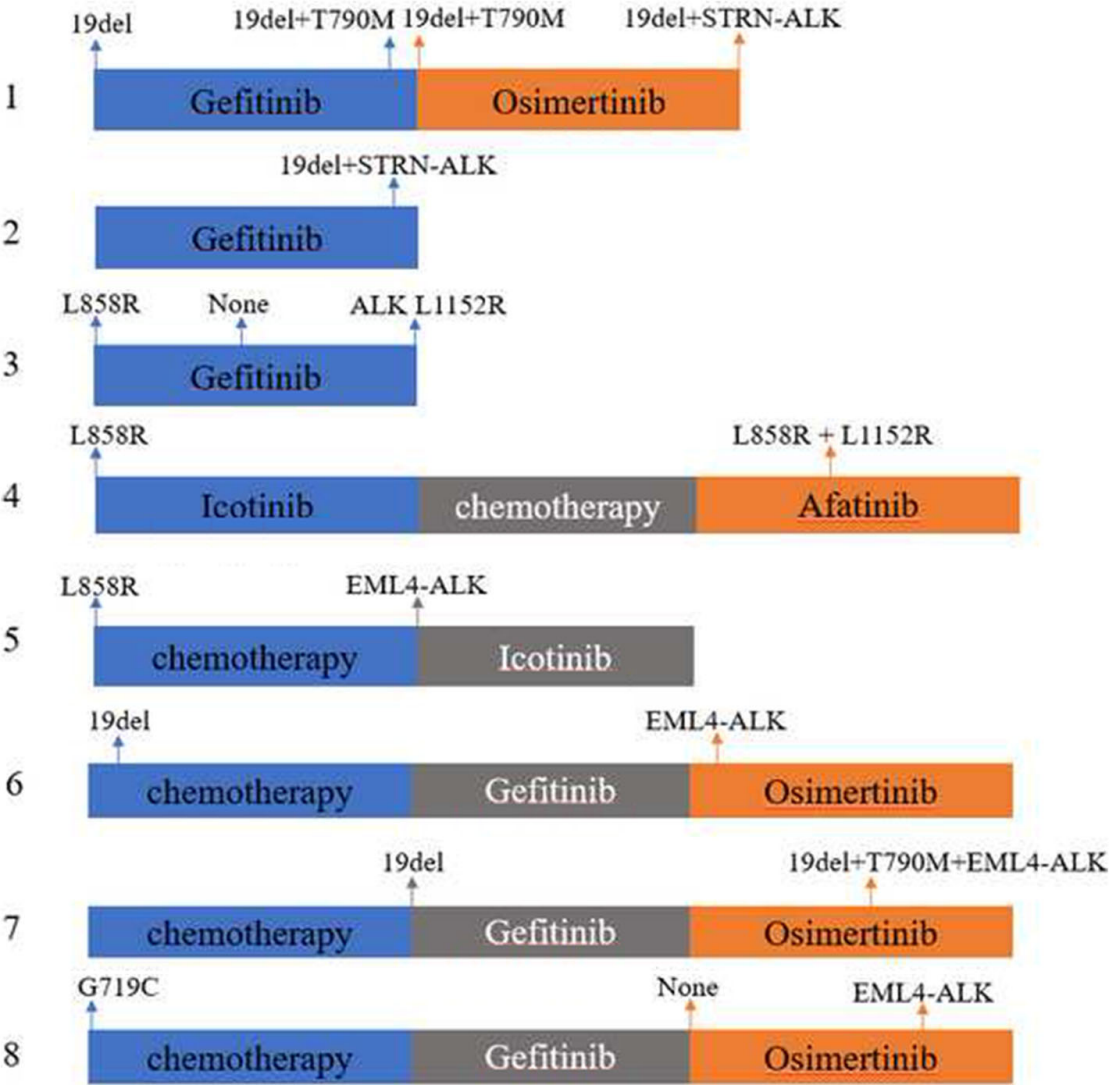
Dynamic monitoring in nine EGFR / ALK co-mutated patients

We performed EGFR and ALK mutation dynamic monitoring in eight of the nine EGFR / ALK co-mutation patients. Eight patients underwent EGFR / ALK dynamic monitoring twice or more. (Fig. 4). Except for one patient (T790M + C797G) who progressed on Gefitinib and subsequently on Osimertinib, the other seven EGFR-TKI resistance patients had no previously known resistance mutations. Three patients had NGS testing before taking EGFR-TKIs. None of them had ALK mutations at that time. Later, one patient (19InDel) gained an STRN-ALK after 15 months on Osimertinib, another (L858R) gained an EML4-ALK after 5 months on Gefitinib, and a third (L858R) gained an L1152R after 10 months on Afatinib. Therefore, in these three patients, ALK mutations were likely developed as resistance mechanisms during EGFR-TKIs therapies. Unfortunately, with no information on ALK status before EGFR-TKI therapies, we cannot tell whether the ALK mutations also occurred during EGFR-TKI therapies in the other five patients. Both STRN-ALK and ALK L1152R were recorded four times in our database, and they concurred with EGFR actionable mutations in 3 and 2 of the 4 records.

## Discussion

EGFR mutations and EML4-ALK translocations have long been considered mutually exclusive [11, 13, 25] . However, there is increasing evidence that concurrent mutations, although rare, do occur [26, 27]. This phenomenon could result from two situations. (1) With tumor heterogeneity, different tumor cell clones separately carry EGFR mutation or ALK rearrangement [28]. (2) The same tumor cell clone carries both an EGFR mutation and an ALK rearrangement [29, 30]. In our study, we identified 9 patients with EGFR / ALK co-mutations and summarized the nine patients’ basic clinical information and treatment prognosis.

Based on our database, the frequency of concurrent EGFR and ALK mutations is relatively low, occurring in only 2.6% of ALK-positive, and 0.2% of EGFR-positive patients. Consistent with our study, Yang et al. [30] showed that the frequency of EGFR/ALK concurrence in NSCLC was 1.3%. However, a study by Liu et al. [31], found the frequency of EGFR/ALK co-alternation to be 5%. To this, we propose that the elevated measurement for EGFR and ALK concurrence resulted from the use of higher sensitivity second-generation sequencing.

Two types of ALK rearrangement (STRN-ALK and ALK L1152R.STRN) were reported to coexist more frequently with EGFR mutations than others [31]. Our study reports for the first time that the ALK rearrangement L1152R may also occur more frequently in combination with EGFR mutations.

There is limited information on the effects of pharmaceutical treatment to these concurrent mutations. A recent study showed EML4-ALK rearrangements could be a rare acquired resistance mechanism following EGFR-TKIs treatment, but there are also studies showing a more common gain of EGFR mutations following ALK-TKI [32].

In our study, a drug-resistant mutation was found after first-line treatment in only one patient (with a T790M mutation). Three patients were found to gain ALK mutations after treatment with EGFR-TKIs. This suggests that the presence of ALK mutation may be related to EGFR-TKI resistance.

In our study, the median PFS for EGFR-TKIs was14.5 months, which is longer than the PFS from phase III clinical trials for first generation EGFR-TKIs [33]. Synchronous or heterochronous EGFR/ALK co-alterations also seems benefit from EGFR-TKIs. However, our study indicates for the first time that ALK L1152R and EGFR concurrent mutations predict shorter EGFR-TKIs PFS.

This study has several limitations. (1) Though we screened over 5000 patients, only 9 EGFR/ALK co-mutations patients were found. Without further screening, we cannot draw a clear conclusion on the clinical characteristics and treatment. (2) Not all EGFR/ALK co-alternation patients were collected at baseline, and some patients lacked detailed treatment records. Therefore, the clinical efficacy of further treatment could not be fully evaluated. (3) All EGFR/ALK co-mutation patients did not receive ALK-TKI treatment, and we will need further experiments to verify our conclusions. Still, our findings do have attractive implications for clinical practice. In conclusion, we found that patients with this condition (except the specific EGFR/ ALK L1152R type) still benefit from EGFR-TKIs. We propose that ALK mutations may be a mechanism of acquired drug resistance of EGFR-TKI treatment. The underlying molecular mechanisms of concurrent mutations require further study.

## Conclusion

In conclusion, we measured the frequency of EGFR and ALK co-alterations and how they may relate to patient clinical characteristics. We propose that ALK mutations can developed as resistance mechanisms during EGFR-TKIs therapies. Most EGFR/ALK co-alteration patients also benefit from first line EGFR-TKIs.

## Supplementary Information

**Additional file 1: Table S1**. The list of 59 gene targeted by NGS. **Table S2**. The list of all mutations detected by NGS.

## Data Availability

The data and materials used and analyzed in the current study are available from the corresponding author on request.

## Abbreviations

TKIs: EGFR-tyrosine kinase inhibitors
PFS: Progression free survival
NSCLC: Non-small cell lung cancer
EGFR: Epidermal growth factor receptor
ALK: Anaplastic lymphoma kinase
NGS: Next Generation Sequencing
ORR: Objective response rate
CR: Complete response
PR: Partial response
SD: Stable disease
PD: Progressive disease
