# Supplementary material for: Genetic and treatment profiles of patients with concurrent Epidermal Growth Factor Receptor (EGFR) and Anaplastic Lymphoma Kinase (ALK) mutations
Source: BMC Cancer. 2021 Oct 15;21:1107. doi: 10.1186/s12885-021-08824-2 (PMC8520304; doi:10.1186/s12885-021-08824-2)
Supplement: Supplementary file 1 — Additional file 1: Table S1. The list of 59 gene targeted by NGS. Table S2. The list of all mutations detected by NGS. [file 12885_2021_8824_MOESM1_ESM.zip › Supplementary table 2 R5.docx]

| P1 | P2 | P3 | P4 | P5 | P6 | P7 | P8 | P9 |
| --- | --- | --- | --- | --- | --- | --- | --- | --- |
| PASK | EGFR | PTPRU | EGFR | TP53 | EML4-ALK | EGFR | EGFR | MSH2 |
| FAT2 | TP53 | DNMT3A | ALK | EML4-ALK | EGFR | TP53 | STRN-ALK | MLH1 |
| EGFR | STRN-ALK | ALK |  | EGFR |  | PIK3CA |  | ROS1 |
| EZH2 |  | ARAP3 |  |  |  | EML4-ALK |  | EGFR |
| TP53 |  | SLX4 |  |  |  |  |  | PTCH1 |
| COL5A3 |  | BRIP1 |  |  |  |  |  | BRCA2 |
| C11orf30 |  | EGFR |  |  |  |  |  | TP53 |
| GAB2 |  |  |  |  |  |  |  | SMARCA4 |
| STRN-ALK |  |  |  |  |  |  |  | EML4-ALK |

Supplementary table 2

The list of all mutations detected by NGS
