# Supplementary material for: Genetic and treatment profiles of patients with concurrent Epidermal Growth Factor Receptor (EGFR) and Anaplastic Lymphoma Kinase (ALK) mutations
Source: BMC Cancer. 2021 Oct 15;21:1107. doi: 10.1186/s12885-021-08824-2 (PMC8520304; doi:10.1186/s12885-021-08824-2)
Supplement: Supplementary file 1 — Additional file 1: Table S1. The list of 59 gene targeted by NGS. Table S2. The list of all mutations detected by NGS. [file 12885_2021_8824_MOESM1_ESM.zip › supplemetal tableR5.pdf]

Supplemental table1 The list of 59 genes NGS targets

ALK	ATM	BRAF	BRCA1	BRCA2	CCND1
CDK4	CDK6	CDKN2A	DDR2	EGFR	ERBB2
FBXW7	FGFR1	KIT	KRAS	MAP2K1	MET
MLH1	MSH2	MSH6	NF1	NRAS	NTRK1
PIK3CA	PMS2	PTCH1	PTEN	RB1	RET
ROS1	SMARCA4	SMO	STK11	TP53	TSC1
TSC2	CYP2D6	UGT1A1	HRAS	IDH1	PDGFRA
AKT1	APC	AR	CTNNB1	ESR1	MTOR
FGFR2	FGFR3	FLT3	SRC	NTRK1	VHL
IDH2	JAK2	MAP2K2	PTPN11	RAF1	
